# Construction and Validation of a Perceived Physical Literacy Instrument for Physical Education Teachers

**DOI:** 10.1371/journal.pone.0155610

**Published:** 2016-05-19

**Authors:** Raymond Kim Wai Sum, Amy Sau Ching Ha, Chih Fu Cheng, Pak Kwong Chung, Kenny Tat Choi Yiu, Che Chun Kuo, Chung Kai Yu, Fong Jia Wang

**Affiliations:** 1 Department of Sports Science and Physical Education, The Chinese University of Hong Kong, Hong Kong SAR, China; 2 College of Sports and Recreation, National Taiwan Normal University, Taiwan, ROC; 3 Department of Physical Education, Hong Kong Baptist University, Hong Kong SAR, China; 4 Department of Recreation and Leisure Industry Management, National Taiwan Sport University, Taiwan, ROC; Nathan Kline Institute and New York University School of Medicine, UNITED STATES

## Abstract

The purpose of this study was to construct and validate a “Perceived Physical Literacy Instrument” (PPLI) for physical education teachers. Based on literature review and focus group interviews, an 18-item instrument was developed for the initial tests. This self-report measure, using a 5-point Likert scale, formed the PPLI and was administered to 336 physical education teachers in Hong Kong. The sample was randomly split, and exploratory and confirmatory factor analyses resulted in a 9-item, 3-factor scale. Exploratory factor analysis (EFA) item loadings ranged from 0.69 to 0.87, and Cronbach’s alpha ranged from 0.73 to 0.76. Confirmatory factor analysis (CFA) showed that the construct demonstrated good fit to the model. The PPLI thus appeared to be reliable and valid to measure the perceived physical literacy of physical education teachers. It is argued that the instrument can be used for both research and applied purposes and potential uses for the instrument in physical education, medical and health settings are discussed.

## Introduction

Since the term physical literacy was introduced, it has globally become a topic of interest in the areas of health and physical education. Experts [1–3] representing different countries have provided insight into the increased acceptance and implementation of the term. Countries such as England and Canada have for several years embraced and extolled the value of this multifaceted concept of physical literacy.[4] In 2015, the Journal of Sport and Health Science published a special issue on physical literacy with 10 articles reviewing the concepts and applicability of physical literacy. [5–14] Indeed, physical literacy has become a major focus of physical education, physical activity and sports and is widely used and recognized in the field. Further, The United Nations Educational, Scientific and Cultural Organization [15] asserts that a quality physical education (QPE) is a core part of school curricula which is important to all students attending school and is particularly salient for those who are responsible for the well-being of students, that is, physical education teachers. Therefore, it is important to develop an instrument which can be used to measure perceived physical literacy for the general public, and particularly the profession that teaches students physical education.

### Physical Literacy

One of the first written definitions of physical literacy was provided by Morrison [16]—“To be physically literate, one should be creative, imaginative, and clear in expressive movement, competent and efficient in utilitarian movement and inventive, versatile, and skillful in objective movement. The body is the means by which ideas and aims are carried out and, therefore, it must become both sensitive and deft” (p. 5). Since then, many similar definitions of physical literacy have been put forth. [2,17–26]

Being credited as one of the leading experts in physical literacy, Whitehead [27] modified her own definition over the years and most recently described physical literacy as “a disposition acquired by individuals encompassing the motivation, confidence, physical competence, knowledge, and understanding that establishes purposeful physical pursuits as an integral element of their lifestyle” (p. 41).

In other words, a physically literate individual is one who embodies the physical nature of movement and uses their experiences and knowledge to interact with the environment.[1,24] Physical literacy educates individuals about their physicality, which not only pertains to ‘being physical’, but encapsulates an embodied understanding of how to be physical by interacting with varied and challenging environments. [1,24,28] In addition, Corbin [29] stressed that “physical literacy provides a foundation for elite sport, public health, and physical education rather than merely being a term used to improve public perceptions” (p. 24).

### Physical Literacy and Physical Education

Penney and Chandler [22] have suggested that physical education is not about teaching children specific activities; rather that it is teaching them specific skills and competencies through the activities. This is dependent on teachers interpreting the curriculum in a way which does not focus on the ‘sport’ of physical education, highlighted through success in learning how to perform in specific activities. Instead, it is to highlight the ways in which necessary skills can be learned through the activities children do. This emphasizes the intended purpose of physical education; that it is for children to develop appropriate skills, understand strategies for moving within a specific environment, and, finally, to understand how this affects their health, rather than providing children with an opportunity to become physically fit through physical education lessons. Thus, it is clear that physical education presents one primary purpose—to develop children’s physical literacy. [24]

The concept of physical literacy can also be explored in the contexts of the child as an active learner, teachers as active practitioners and schools and the curriculum as providing active contexts. [20] The significance of physical literacy is considered to be an integral part of the education of the whole child, being made up of a wide range of ‘intelligences’. Armstrong [30] suggested that “the way exercise is presented to children may have important implications for future activity patterns and consequently for their health and well-being as adults”.

Recent researches suggest that physical education curriculum should have a physical literacy approach including fundamental movement skills, [31] and the development of physical literacy is critical to long-term health. [32] Physical literacy is a significantly important goal of physical education. An understanding of physical literacy would help one appreciate the special nature of physical education. This does not pertain specifically to teaching children and young people to play sports; nor is it purely about finding those with the potential to become elite sportsmen. Physical education is about encouraging every child and young person to become a lifelong participant in physical activity and supporting every child and young person on their physical literacy journey.

In this sense, the role of physical education teachers is important in the implementation of physical literacy in physical education so that future generations have the “motivation, confidence, physical competence, knowledge, and understanding to value and take responsibility for maintaining purposeful physical pursuits and activities throughout the lifecourse”. [26]

Since physical literacy is the ultimate goal of physical education, the definitions of physical literacy are intended to assist physical education teachers to implement quality physical education curricula, extra-curricular activities and health promotion programs etc., which are designed to foster the development of physically literate students. Part of the highlights of physical literacy is the development of students’ motivation and confidence. However, it is understood that motivation and confidence cannot be ‘taught’ and focus should be placed more on ‘learning’. Teaching is designed to provide learning opportunities through which abilities can be nurtured. Therefore, it is important for physical education teachers to understand the concept of physical literacy so that students of today are not only better prepared to live a healthy life, but are better equipped to do so in ways that assist others, are respectful of the environment, and in ways that have the potential to generate new and innovative ideas. [2]

To conclude, the above literature review has led us to the following three mutually reinforceable key attributes of a physically literate individual: 1) motivation, 2) confidence and physical competence, and 3) interaction with environment. [26]

Therefore, given the importance of physical literacy in the above key attributes, the purpose of this study was to develop and then analyze the properties of a survey instrument–the Perceived Physical Literacy Instrument (PPLI), which can be used to investigate physical education teachers’ self-perception of physical literacy.

## Methods

### Sample and Procedures

The approval for the use on human subjects was obtained from the University Survey and Behavioural Research Ethics Committee at the Faculty of Education, the Chinese University of Hong Kong. A total of 337 physical education teachers (162 male; 173 female; 2 unknown gender) with teaching experience ranging from 1 to 36 years who taught in primary school (N = 125) and secondary school (N = 210) attended the annual continuous professional development conference in Hong Kong were asked to complete the questionnaire in June, 2015. Written informed consent for participation in the study was obtained from participants. Trained research staff was responsible for distributing and collecting the questionnaire. They also answered any questions raised by the participants. The average range of questionnaire completion time was 8–10 minutes.

### Designing the Physical Literacy Survey

The original item pool was constructed from a comprehensive literature review on the concept of physical literacy between 1994 and 2015, followed by three focus group interviews which lasted an average of 90 minutes per interview. Eleven Hong Kong Chinese physical education teachers with teaching experience ranging from 3 to 18 years were interviewed on three separate occasions. The focus group participants were all physical education subject specialists and came from primary (N = 5) and secondary (N = 6) schools in Hong Kong. This number was appropriate, as focus group sizes generally range from 4 to 12. [33] In the focus group discussions, participants were given opportunities to share their perception on physical literacy and to provide diverse experiences on how physical literacy impacts their students’ lifestyles. The researchers used reviewed literature to guide the focus group interviews that helped to identify some key attributes of physical literacy and relationships between all attributes such as “sense of self and self-confidence”, “self-expression and communication with others” and “knowledge and understanding” [26] for the development of the instrument.

The interviews were audio-taped and transcribed by the researchers. Content analysis was used to identify codes in an iterative process. [34] The researchers discussed the codes and their relationships in order to facilitate analysis for drafting different items of the instrument. The role of codes was to distinguish overall themes in which more specific patterns can be interpreted. After drafting and revising items, participants from the focus group were asked to complete an 18-item version of the instrument. They responded to the items, evaluated their clarity, and provided feedback on the response scale. The feedback suggested slight changes to 2 items and that the instrument could be achieved by using a 5-point Likert scale anchored by 1) “strongly disagree” and 5) “strongly agree” with a midpoint 3) “no comment”.

A second (revised) version of the instrument was then sent to a panel of four experts who were teaching and doing research in the area of sport science, physical education, health education and instrument development in local and regional universities. They were invited to evaluate the initial instrument for item wording, instrument length, clarity of the statement and response format. Revisions were made based on their feedback with a few items rewritten. There were no suggested items being added by the experts relating to the construct. The initial instrument was well received and commented as useful. A full outline of the items of the instrument which contained 18 questions on the understanding of physical literacy is shown in Table 1.

**Table 1:** Items of the instrument contained 18 questions on understanding of physical literacy.

1.	I possess adequate fundamental movement skills
2.	I am physically fit, in accordance with my age.
3.	I am able to apply learnt motor skills to other physical activities
4.	I have a positive attitude and interest in sports
5.	I appreciate myself or others doing sports
6.	I am able to apply PE knowledge in the long run
7.	I possess self-management skills for fitness
8.	I possess self-evaluation skills for health
9.	I am willing to do sports for better health
10.	I have strong communication skills
11.	I have strong social skills
12.	I am confident in wild/natural survival
13.	I am capable in handling problems and difficulties
14.	I have a mindset for lifelong sports
15.	I can turn doing sports into an on-going habit of life
16.	I establish friendship through sports
17.	I am aware of the benefits of sports related to health
18.	I aspire to know the current sports trend

All items are measured on a 5-point Likert scale (1: strongly disagree to 5: strongly agree).

### Data Analysis

Firstly, the research team used the expectation-maximization (EM) algorithm [35] for estimating missing values in returned questionnaires. There were 1.8% of the overall values missing (6 cases), with 1 missing case excluded from the analysis as over 50% of their data fields were missing. In the 5 other missing cases, only a trivial number of values were missing and the missing pattern was random. As such, it was reasonable to hypothesize that the remaining cases were representative of the entire sample and those missing cases were the same as the non-missing cases in terms of the analysis being performed. [36] The research team therefore implemented an ad hoc deletion of missing data before proceeding to the next step.

Samples (N = 336) were randomly allocated via a computer-generated randomization sequence (GraphPad Software, Inc.) into two groups with the ratio 1:2 (135 cases in set 1 and 201 cases in set 2) in which the sample size was satisfied with the minimum amount of data. [37] Data from the first half of the split sample (N = 135) were entered into the IBM SPSS 21 for Windows program. The research team then conducted an exploratory factor analysis (EFA).

To confirm the factor structure obtained in the first half of the split sample (N = 201), the research team conducted a confirmatory factor analysis (CFA) using structural equation model (SEM) techniques for the second half of the split sample. CFA was used as a critical step to refine the instrument and identify factor structure within understood physical literacy. The data were analyzed using AMOS 22.

## Results

The results revealed a three-factor scale, with Eigenvalues greater than 1.0, accounting for 68% of the total variance. Factor loadings ranged from .69 to .87, and 9 items were retained. Internal consistency of each factor was assessed using Cronbach’s alpha; these values ranged from .73 to .76, all meeting the criterion level of .70. [38] Moreover, the values of item-total correlations ranged from .40 to .68.

### Results of Exploratory Factor Analysis (EFA) on Subset 1

The research team performed EFA for the first subset on the 18 items (see Table 1). The optimal number of factors was determined by latent root criteria (eigenvalues > 1.0, the Kaiser’s criterion K1). The Kaiser-Meyer-Olkin (KMO) index of sampling adequacy appeared to be significant (.83). Bartlett’s test of sphericity proved that correlations between items were large enough for conducting a principal components analysis (PCA) (p < .001; our model 0.000). [37]

Nine out of 18 items were deleted after initial factor analysis and this resulted in the final three factors as shown in the pattern matrix in Table 2.

**Table 2:** Factor structures by Exploratory Factor Analysis and Reliability (N = 135).

Sign	Items	F1	F2	F3	Total Variance explained	Item-total Correlation (subscales)	Item-total Correlation (full scale)	Scale Alpha
PL4	I have a positive attitude and interest in sports	**0.69**	0.31	0.22		0.55	0.58	0.73
PL5	I appreciate myself or others doing sports	**0.87**	0.02	0.07		0.58	0.40	
PL17	I am aware of the benefits of sports related to health	**0.73**	0.18	0.19		0.52	0.49	
PL2	I am physically fit, in accordance to my age	0.03	**0.83**	0.06		0.51	0.42	0.76
PL7	I possess self-management skills for fitness	0.21	**0.78**	0.26		0.65	0.60	
PL8	I possess self-evaluation skills for health	0.31	**0.70**	0.28		0.62	0.64	
PL11	I have strong social skills	0.20	0.10	**0.78**		0.55	0.52	0.76
PL12	I am confident in wild/natural survival	0.07	0.19	**0.80**		0.68	0.51	
PL13	I am capable in handling problems and difficulties	0.21	0.22	**0.78**	67.95%	0.64	0.62	

*Note*. The cronbach’s alpha for full scale is 0.82.

With 3 variables per factor, three factors were found in the final analysis generally confirming the differentiation between the categories of understanding of physical literacy that were based on previous literature. Items with communality of less than 0.40 were removed from the analysis [39] and the PCA was computed again with all items greater than 0.60 in our final model. A cross loading item was defined as an item that loads at 0.32 or higher on two or more factors. [40] Crossloading items were dropped from the analysis and the PCA was reconducted.

In addition, to assess the fit of the factor models, the research team examined the differences between the model-based correlations and the observed correlations. Our model showed that 33% of the residuals was greater than 0.05. [41] In terms of the internal consistency of the three factors, the research team used Cronbach’s α for the entire data set (see Table 2), and all factors showed sufficient to good (>0.7).

### Results of Confirmatory Factor Analysis (CFA) on Subset 2

**Fig 1:**
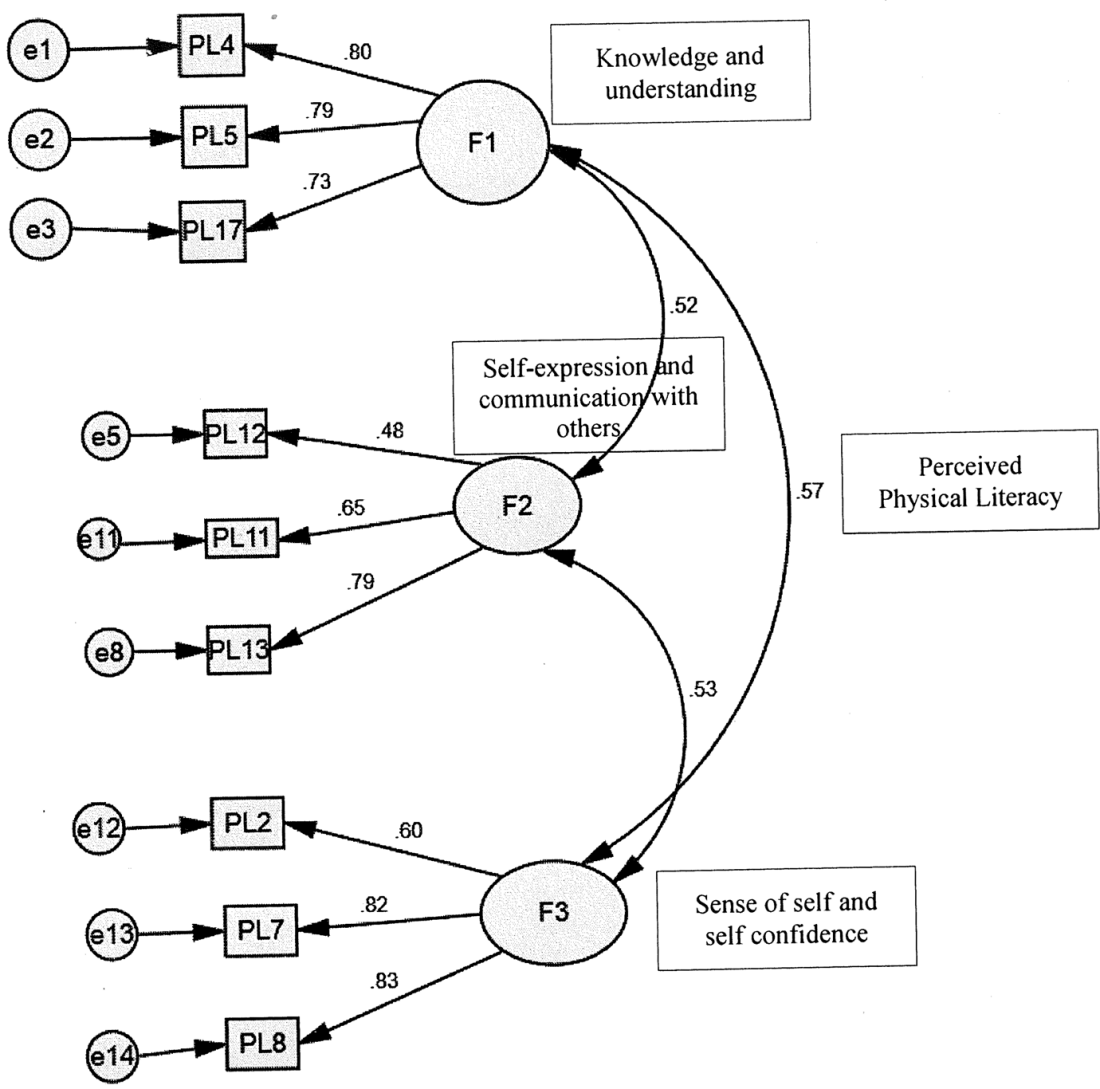
Factor structure and standardized factor loading on perceived physical literacy items.

Based on the EFA results, CFA was performed with IBM SPSS-Amos 22 to cross-validate and confirm the three-factor structure derived in the analysis using the first subset. The research team assessed the factorial validity of the three scales by confirmatory factor analysis. The factor loading of all items (Fig 1), above the standard of .45, [42] ranged from .48 to .83, revealing the factor validity of the measurement was satisfactory. The goodness-of-fit test on the model was assessed using chi-square, comparative fit index (CFI), [43] and root-mean-square error of approximation (RMSEA). [44] Non-significant chi-squares are considered as an acceptable model fit. [45] Values which are greater than 0.90 are considered as an acceptable model fit for the CFI. [46] The RMSEA is often considered one of the most valuable fit indices in SEM. Hu and Bentler [47] indicated a value of .06 would suggest a good fit. Final fit statistics were all adequate as follows: chi-square (p>.05), CFI = .95, RMSEA = .038.

## Discussion

This study conceptualized a construct, and developed and validated an instrument to measure perceived physical literacy for physical education teachers. Results showed that the instrument has good validity and reliability of a scale designed to measure physical education teachers’ perception of their own physical literacy in terms of their sense of self and self-confidence, self-expression and communication with others, and knowledge and understanding. Items 2, 7 and 8 stated: “I am physically fit, in accordance with my age”, “I possess self-management skills for fitness” and “I possess self-evaluation skills for health”; they expressed that a physically literate individual should have a better sense of self and self-confidence. Items 11, 12 and 13 listed: “I have strong social skills”, “I am confident in wild/natural survival” and “I am capable in handling problems and difficulties”; which showed that a physically literate individual should possess better self-expression and communication skills. Items 4, 5, 17 stated: “I have a positive attitude and interest in sports”, “I appreciate myself or others doing sports” and “I am aware of the benefits of sports related to health”; they depicted that a physically literate individual should have a better knowledge and understanding of the benefits of being physically active. The above three attributes echoed with the other three attributes which are the kernel of the concept of physical literacy proposed by Whitehead [26]. Motivation, confidence and physical competence, and interaction with the environment are the other attributes that are mutually reinforced with sense of self and self-confidence, self-expression and communication with others, and knowledge and understanding. [26]

The research team believed that a valid perceived physical literacy instrument (PPLI) for physical education teachers or other professionals and populations has yet to be developed. The results of this empirical study appeared to be consistent with the attributes in the literature which are presently dominant and persistent in international literature [3,9,24–26,32]: 1) motivation, 2) confidence and physical competence, 3) interaction with the environment, 4) sense of self and self-confidence, 5) self-expression and communication with others, and 6) knowledge and understanding.

There are several practical implications. First, it should be highlighted that this instrument is an appropriate testing tool for identifying an individual’s perceived physical literacy. As such, the instrument can be considered a measure of generalized physical literacy which is particularly useful for students because they should be involved in different physical activities that are aimed to develop their fundamental movement skills and internalize their physical literacy. [26] Second, this instrument can be used for assessing the effectiveness of intervention programs that are important to physical education teachers’ continuous professional development and students’ learning outcomes in physical education lessons. In particular, a comprehensive physical literacy assessment of engagement in physical activity, physical competence, motivation and confidence, and knowledge and understanding related to a physically active lifestyle for children aged 8 to 12 years. [48] Third, from public health perspective, physical educators and related professionals in medical and health settings should also consider using this instrument to measure perceived physical literacy in different populations. As there is an increased awareness of physical literacy levels, this in turn has potential to improve the quality of people's healthy active lifestyle. Results will provide policy makers, physical educators and health professionals with an understanding of the needs of being physically active throughout one's lifecourse in different populations. [26], [49]

Notably, there are limitations to this study. First, similar with many instrument development studies, this study did not include a random sample of physical education teachers. The participants recruited in this study may have more understanding of what physical literacy means. Second, measurement of this study was based on self-report, which may have been prone to response and information bias, especially as physical education teachers were more proactive in their response to the invitation to participate in this study at the commencement of a continuous professional development conference. Third, this study was cross-sectional in character which cannot demonstrate causality between factors associated with perceived physical literacy and the temporal stability of the current instrument was unwarranted. By considering above limitations, future research focusing on longitudinal inquiry is recommended to better examine the factor model’s invariance of physical literacy for physical education teachers over time. Furthermore, in order to enhance the robustness, flexibility, and generalizability of physical literacy instrument, further testing the validity and reliability of the instrument’s application in different populations may be needed. In doing so, not only the temporal stability and the cross-validation of the physical literacy instrument can be met, but the understanding of the relationship and causal effects of physical literacy of physical education teachers in different school settings or indeed in various countries, cultures and environments will be extended. [50]

Further research should also investigate the relationship between perceived physical literacy for physical education teachers and different measures of psychological constructs such as self-efficacy, self-concept and motivation. The influence of changes in physical education teachers’ perception of physical literacy and self-efficacy on students’ motives in undertaking physical activity is also a potential research point to explore students’ learning outcomes of physical education lessons.
